# Rats deficient in α-galactosidase A develop ocular manifestations of Fabry disease

**DOI:** 10.1038/s41598-019-45837-1

**Published:** 2019-06-28

**Authors:** James J. Miller, Kazuhiro Aoki, Christopher A. Reid, Michael Tiemeyer, Nancy M. Dahms, Iris S. Kassem

**Affiliations:** 1 Department of Biochemistry, Medical College of Wisconsin, Milwaukee, WI USA; 2 Complex Carbohydrate Research Center, University of Georgia, Athens, GA USA; 3 Department of Cell Biology, Neurobiology, and Anatomy, Medical College of Wisconsin, Milwaukee, WI USA; 4 Department of Ophthalmology and Visual Sciences, Medical College of Wisconsin, Milwaukee, WI USA

**Keywords:** Hereditary eye disease, Glycobiology

## Abstract

Fabry disease is an X-linked lysosomal storage disease caused by deficiency of α-galactosidase A. Ocular findings, such as cornea verticillata, cataracts, and retinal vascular tortuosity, serve as important diagnostic markers. We aimed to evaluate ocular phenotypes in α-galactosidase A-deficient (Fabry) rats and hypothesized that these rats would manifest ocular signs similar to those observed in patients. Slit lamp biomicroscopy was used to evaluate the cornea and lens, and retinal vasculature was examined by fluorescein angiography in WT and Fabry rats. Mass spectrometry was used to characterize and quantify ocular glycosphingolipids, and histology and electron microscopy revealed the location of the glycosphingolipid storage. We found that Fabry rats developed corneal and lenticular opacities to a statistically greater degree than WT rats. Retinal vascular morphology did not appear grossly different, but there was vascular leakage in at least one Fabry rat. Fabry rat eyes accumulated substrates of α-galactosidase A, and these α-galactosyl glycoconjugates were found in corneal keratocytes, lens fibers, and retinal vascular endothelial cells. Electron-dense lamellar inclusions were observed in keratocytes. Because Fabry rats recapitulate many ocular phenotypes observed in patients, they can be used to study disease pathogenesis and determine whether ocular findings serve as noninvasive indicators of therapeutic efficacy.

## Introduction

Fabry disease (OMIM #301500) is an X-linked lysosomal storage disease caused by a deficiency in α-galactosidase A (α-Gal A) activity. As a result, substrates of α-Gal A, predominantly glycosphingolipids, accumulate and lead to cellular dysfunction. The prevalence of Fabry disease is estimated at approximately 1:3000 to 1:8500 births^1,2^. Patients have a shorter and poorer quality of life from renal failure, cardiac dysfunction, cerebrovascular disease, gastrointestinal symptoms, and chronic pain (reviewed in^3^). Ocular findings are common and correlate with disease severity using the Fabry Outcome Survey Mainz Severity Score Index^4,5^. The most common ocular finding is cornea verticillata, which is found in approximately 85% of males and 75% of females; lenticular opacities and vascular tortuosity of the conjunctival, retinal, or choroidal vessels are also observed (reviewed in^6^). The most vision-threatening complication is retinal vascular occlusion, which can cause sudden blindness and neovascularization^7–9^. Therapeutic interventions for Fabry disease are targeted at improving the quality of life and extending lifespan.

One FDA-approved treatment for Fabry disease is enzyme replacement therapy (ERT) where recombinant α-Gal A protein is infused biweekly. The regimen is costly and has the possibility of triggering life-threatening immune reactions^10^. Because the response to ERT is variable, much research effort is placed on the development of new therapies. Recently, the FDA approved migalastat, a small-molecule chaperone indicated for patients with amenable mutations in the gene encoding α-Gal A^11,12^. However, patients with disease non-amenable mutations are unable to benefit from chaperone monotherapy. Therefore, the development of improved therapeutic interventions for all patients is greatly needed.

α-Gal A knockout (KO) mouse models have been important in evaluating therapies to diminish glycosphingolipid storage^13,14^, but the utility of the Fabry mouse in studying eye pathology is limited by the fact that ocular phenotypes are not robustly documented in Fabry mice. We previously generated an α-Gal A KO (Fabry) rat model, which demonstrates pain, cardiac, and renal phenotypes commonly observed in patients^15,16^. In this study, we evaluated the corneal, lenticular, and retinal phenotypes of Fabry rats. We found that Fabry rats develop corneal and lenticular opacities. While there was no overt evidence of retinal tortuosity, Fabry rat eyes accumulate α-galactosyl glycosphingolipids in keratocytes, lens fibers, and retinal vascular endothelial cells. To our knowledge, this is the first study that extensively characterizes ocular phenotypes in an animal model of Fabry disease. Because Fabry rats develop multiple ocular phenotypes similar to those observed in patients, they can be used to better investigate mechanistic pathways of the ocular pathology and the effects of existing and new therapies.

## Methods

### Animals

All rat procedures were approved by the IACUC at Medical College of Wisconsin (Milwaukee, WI, USA) and were carried out in accordance with the recommendations in the *Guide for the Care and Use of Laboratory Animals*. As previously reported, the Fabry rat model (Rat Genome Database symbol: *Gla*^*em2Mcwi*^) was generated using CRISPR/Cas9 technology to disrupt the rat *Gla* gene in the Dark Agouti strain^15^. All rats studied in this report were obtained from the existing Fabry rat colony at Medical College of Wisconsin. In this report, *Gla*^+/0^ males are referred to as WT, and *Gla*^−/0^ males are referred to as KO. *Gla*^+/+^, *Gla*^+/−^, and *Gla*^−/−^ females are referred to as WT, heterozygous (HET), and KO females, respectively.

### Slit lamp biomicroscopy

**Figure 1:**
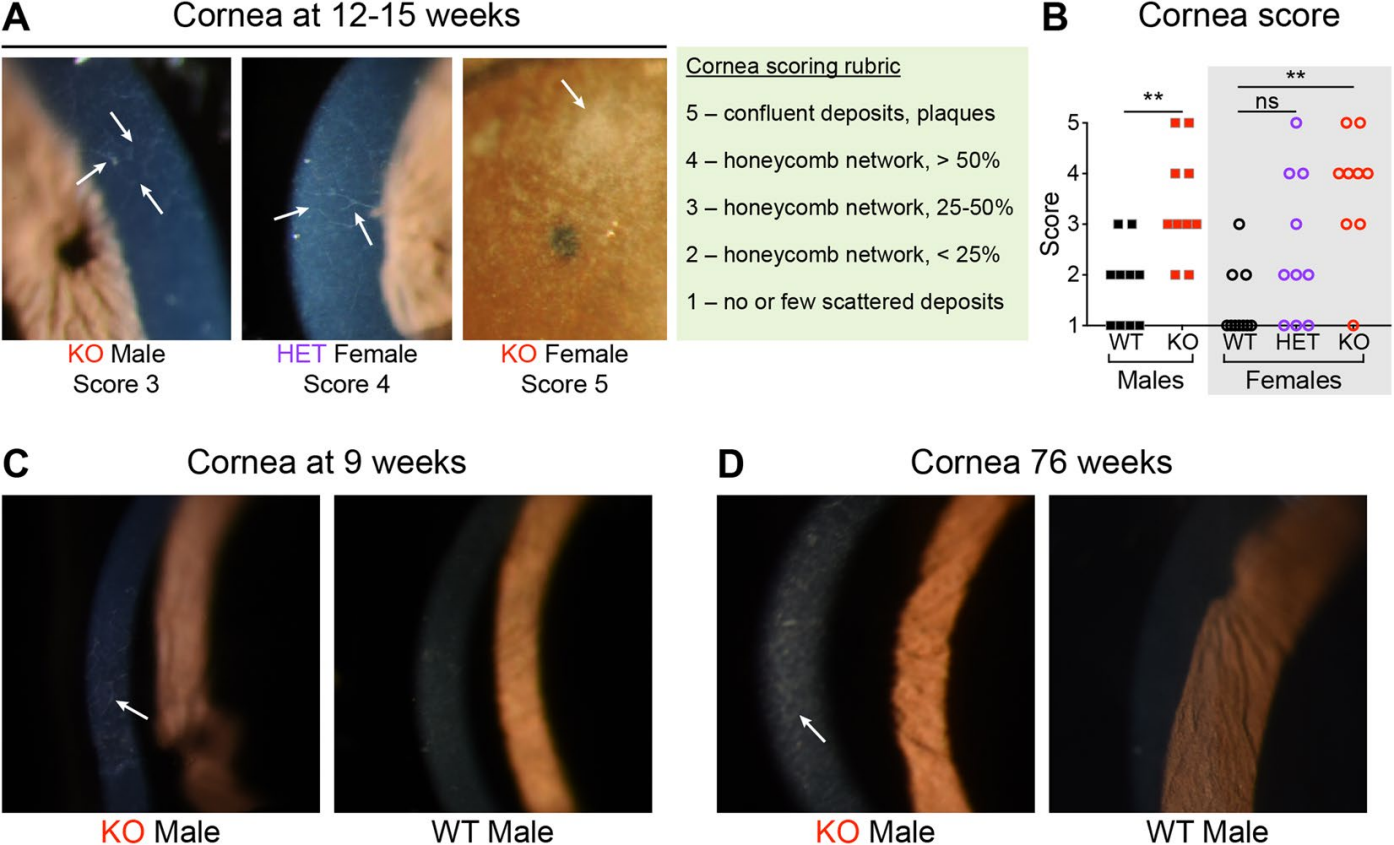
Fabry rats develop corneal opacities. (**A**) Shown are representative slit lamp images of rat corneas at 12–15 weeks of age. The arrows indicate opacities. The corneal opacity scoring rubric is shown in the light green box, and assigned scores are shown below the representative images. (**B**) Corneal opacity scores are plotted from 10 WT males, 10 KO males, 10 WT females, 10 heterozygous (HET) females, and 9 KO females at 12–15 weeks of age. Male median scores were compared using a Mann-Whitney test, and female median scores were compared using a Kruskal-Wallis test and Dunn’s multiple comparison test; ns = not significant (*P* ≥ 0.05) and **P < 0.01. Representative slit lamp images of rat corneas at (**C**) 9 weeks (n = 2 WT males, n = 2 KO males) and (**D**) 76 weeks (n = 2 WT males, n = 3 KO males) are also shown.

**Figure 2:**
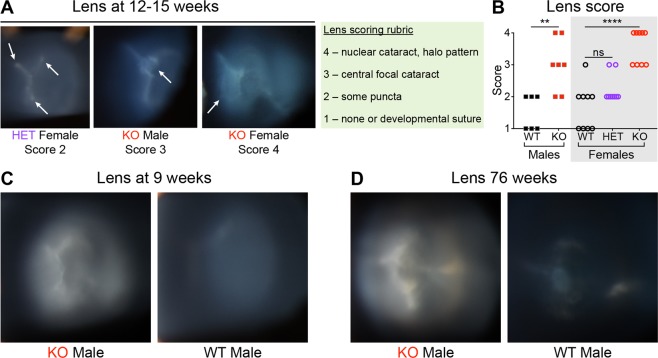
Fabry rats develop lenticular opacities. (**A**) Shown are representative slit lamp images of rat lenses at 12–15 weeks of age. The lens opacity scoring rubric is shown in the green box, and assigned scores are shown below the representative images. White arrows indicate opacities. (**B**) Lens opacity scores are plotted from 6 WT males, 7 KO males, 9 WT females, 9 heterozygous (HET) females, and 9 KO females at 12–15 weeks of age. Male median scores were compared using a Mann-Whitney test, and female median scores were compared using a Kruskal-Wallis test and Dunn’s multiple comparison test; ns = not significant (*P* ≥ 0.05), **P < 0.01, and ****P < 0.0001. Representative slit lamp images of rat lenses at (**C**) 9 weeks (n = 2 WT males, n = 2 KO males) and (**D**) 76 weeks (n = 2 WT males, n = 3 KO males) are shown.

Rats were anesthetized with isoflurane, and their eyes were examined with a slit lamp biomicroscope (Topcon Medical Systems, Oakland, NJ, USA; model SL-D8Z) equipped with a camera (Nikon, Melville, NY, USA; D810 36.3MP DSLR). Corneal examination was performed first, followed by dilation with 1% tropicamide (Akorn, Lake Forest, IL) to allow for examination of the entire lens. Cornea and lens images were randomized and scored post-hoc by a board-certified ophthalmologist in a blinded manner using rubrics developed by our laboratory (Figs 1A and 2A, green boxes).

### Fluorescein angiography

In order to evaluate the retinal vasculature, fluorescein angiography studies were performed. Rats were anesthetized with isoflurane, and their eyes were dilated with 1% tropicamide (Akorn, Lake Forest, IL). Rats were then placed on a platform and imaged with a confocal scanning laser ophthalmoscope (Heidelberg Engineering, Heidelberg, Germany). A custom-made contact lens was applied to the surface of the cornea to reduce desiccation and improve image quality. The camera was aligned to the optic nerve. 10% AK-Fluor (100 mg/ml stock, Akorn, Lake Forest, IL) was injected subcutaneously at a concentration of 0.1 mg/kg. High resolution fluorescent images were recorded using a 486 nm excitation laser with 502 to 537 nm band pass emission filters.

### Glycosphingolipid isolation, mass spectrometry, and thin layer chromatography

Glycosphingolipids were extracted from whole rat eyes (one eye per rat), analyzed by high-performance thin layer chromatography, and quantified by nanospray ionization-mass spectrometry as previously described^15^. For quantification, glycosphingolipids were normalized per whole eye (pmol/eye).

### Histology and electron microscopy

Histology and electron microscopy studies were performed as previously described^15^, including the following modifications for isolectin B4 staining: enucleated rat eyes were fixed in 1% formalin/1.25% glutaraldehyde, as this was found to be an ideal eye fixative^17^. After fixation for approximately 72 hours, the eyes were dehydrated and paraffin embedded. Paraffin sections were cut at 4 µm, placed on poly-L-lysine slides, and rehydrated. The slides were then subjected to antigen retrieval and lectin staining using biotinylated isolectin B4 (Vector Laboratories, Burlingame, CA, USA) at 5 µg/ml followed by standard streptavidin-HRP and 3,3′-diaminobenzidine detection. The stained sections were scanned using a NanoZoomer 2.0-HT digital slide scanner (Hamamatsu, Hamamatsu City, Japan).

### Statistics

Graph Pad Prism 7 software was used to perform all statistical analyses. A Mann-Whitney U test was used to compare male median severity scores, and a Kruskal-Wallis test followed by Dunn’s multiple comparisons was used to compare female median severity scores. The level of significance was set at *P* < 0.05, where ns = not significant (*P* ≥ 0.05), *P < 0.05, **P < 0.01, ***P < 0.001, and ****P < 0.0001.

## Results

Most patients with Fabry disease develop ocular manifestations, and cornea verticillata is a well-known sign. We sought to document whether Fabry rats also develop a corneal phenotype using slit lamp biomicroscopy. Eyes from male (WT and KO) and female (WT, HET, KO) rats were imaged at 12 to 15 weeks of age, and anterior corneal opacities were observed in some rats. These lesions developed in a fine honeycomb pattern in which the opacities formed a closed loop of anterior linear deposits starting superiorly and progressing downward with increasing severity (Fig. 1A). This morphology was distinct from the normal, occasional linear peripheral opacities seen in WT rats. In some rats, the opacities coalesced into thickened, anterior corneal plaque-like lesions (Fig. 1A). We scored the opacities in a blinded and randomized fashion and found that KO male and female rats had higher median severity scores compared to WT males and females, respectively (Fig. 1B). HET female median opacity scores were the most variable and not statistically different from the WT females (Fig. 1B). Further, the corneal opacities were present in KO rats as young as 9 weeks and as old as 76 weeks (Fig. 1C,D, respectively). Therefore, although not the typical whorl-like opacities characteristic of cornea verticillata, our slit lamp imaging studies reveal that Fabry rats develop corneal opacities in a characteristic pattern that can be scored, which is reflective of opacity severity.

Because cataracts are a common finding in patients with Fabry disease, we also observed lens opacities in the same cohort of 12- to 15-week-old rats. The rat lenticular findings existed on a spectrum from no opacities (fine developmental lens suture lines were often apparent and considered normal) to dense nuclear cataracts with concentric, onion-skin-like halo patterns (Fig. 2A). The intermediate lenticular phenotypes included opaque punctate opacities approximating the lens developmental suture or a small central nuclear cataract (Fig. 2A). After randomized and blinded scoring, we found that the KO males and females had greater median severity scores compared to those of WT rats (Fig. 2B). As with the corneal phenotype, the median severity score of HET females was not statistically different from that of WT females (Fig. 2B). Lenticular opacities were observed in younger (9 week) and older (76 weeks) KO rats as well (Fig. 2C,D, respectively). In summary, like patients with Fabry disease, male and female Fabry rats develop lenticular opacities to a significantly greater degree than WT controls.

**Figure 3:**
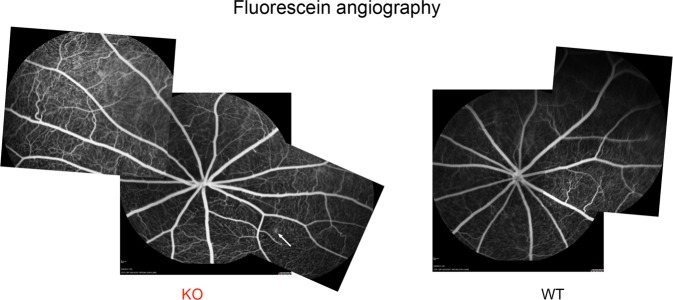
Minimal differences are observed between WT and Fabry rat retinal vasculature. WT and KO rats were subjected to fluorescein angiography, where 0.1 mg/kg fluorescein was injected subcutaneously. Representative images are shown from 10 KO and 9 WT rats at approximately 55–80 weeks of age. The KO image is from a male rat at 57 weeks, and the WT image is from a male rat at 63 weeks. In the KO image, the arrow indicates evidence of vascular leakage.

In addition to corneal and lenticular opacities, patients with Fabry disease can exhibit abnormalities in retinal vasculature, including retinal vessel tortuosity and ischemia. Thus, we used fluorescein angiography to view the retinal vessels in Fabry rats. We observed no obvious tortuosity in KO rats (n = 10) compared to WT rats (n = 9) (Fig. 3, representative images). However, we did observe one KO rat with signs of retinal vascular leakage (Fig. 3, arrow). While we found that Fabry rats develop corneal and lenticular opacities, retinal vessel tortuosity in Fabry rats was not appreciated when assessed by fluorescein angiography.

**Figure 4:**
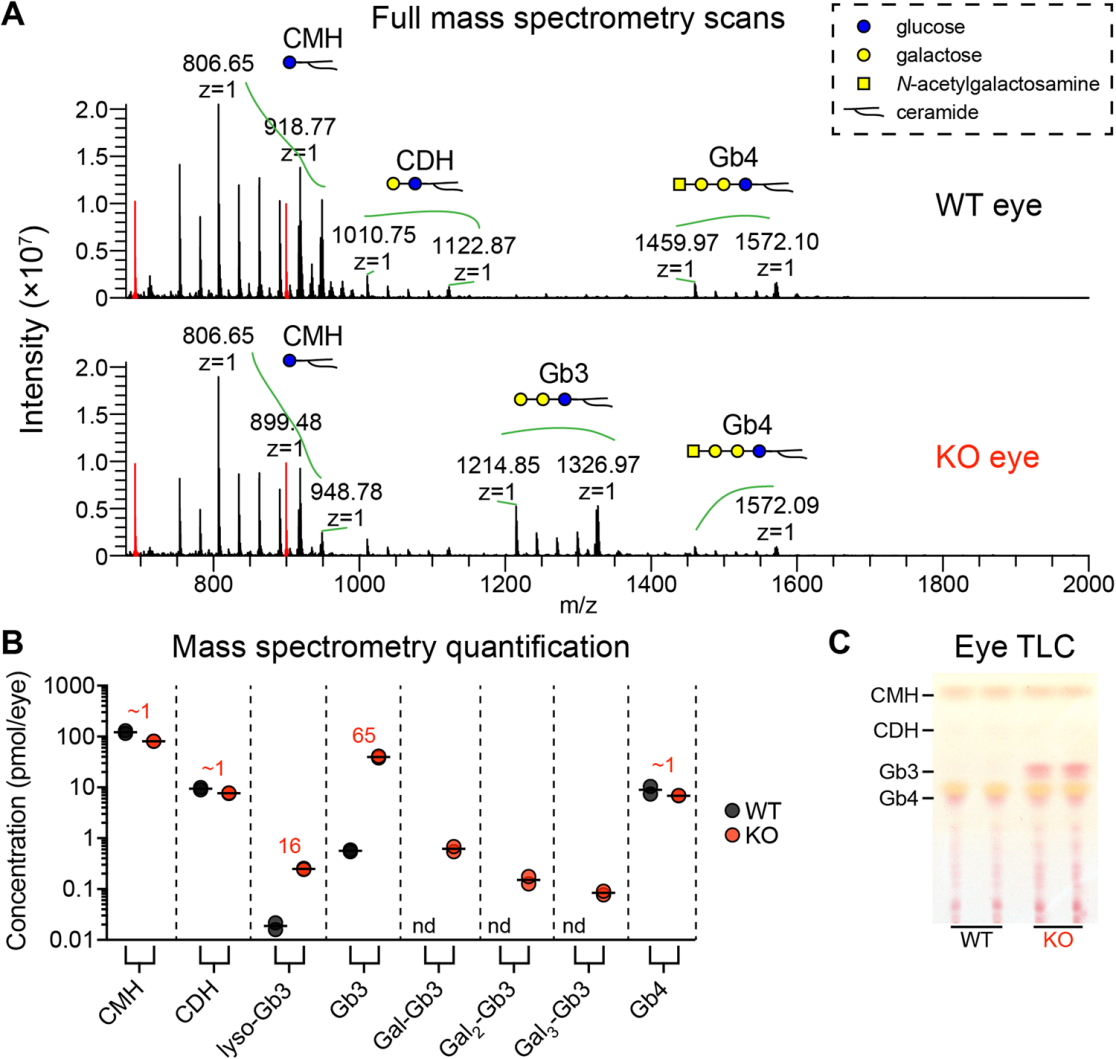
Fabry rat eyes accumulate globotriaosylceramide and related glycosphingolipid biomarkers. (**A**) Glycosphingolipids (GSLs) were extracted from whole rat eyes (2 WT and 2 KO males, 13 weeks) and analyzed by nanospray ionization-mass spectrometry. Representative full mass spectrometry scans are shown. Monosaccharides are represented using the Symbol Nomenclature for Glycans (dashed box). Red peaks represent quantification standards added in equal amounts to all samples. (**B**) Eye GSLs were quantified from the full mass spectrometry scans (note the y-axis is a log scale). Plotted are each individual data point (n = 2 WT, n = 2 KO) with means depicted by the horizontal line. For each GSL, the mean KO concentration fold increase above mean WT concentration is shown (red numbers). Some GSLs were not detected (n.d.) in WT eyes. (**C**) GSLs from rat eyes (2 WT and 2 KO males, 13 weeks) were separated by thin layer chromatography (TLC) using a solvent system of chloroform/methanol/water (60:40:10, *v/v/v*) and visualized by an orcinol-sulfuric acid reagent. The migration locations of the GSL standards are shown to the left of the plate. Abbreviations: ceramide monohexoside (CMH), ceramide dihexoside (CDH), globotriaosylceramide (Gb3), globotetraosylceramide (Gb4), globotriaosylsphingosine (lyso-Gb3), galactose (Gal).

Similar to our previous studies on Fabry rat pain, cardiac, and renal phenotypes^15,16^, we next aimed to characterize the ocular phenotypes at the molecular and cellular levels. Given that patients with Fabry disease are deficient in α-Gal A, they accumulate glycosphingolipids terminated with galactose (Gal) residues in an α-linkage. We profiled the glycosphingolipids extracted from rat eyes using nanospray ionization-mass spectrometry. On full mass spectrometry scans, signals corresponding to the α-galactosyl glycosphingolipid, globotriaosylceramide (Gb3), were readily observed in the spectra from KO but not WT eye (Fig. 4A). When quantified, we found that Gb3 and globotriaosylsphingosine (lyso-Gb3) were elevated at 65- and 16-fold in KO eye compared to WT eye (Fig. 4B). Further, polygalactosylated-Gb3 species, including Gal-Gb3, Gal_2_-Gb3, and Gal_3_-Gb3, were detectable in KO rat eye, but were undetectable in WT rat eye. There were no differences in control glycosphingolipids that do not contain terminal α-galactose residues, such as ceramide monohexoside, ceramide dihexoside, and globotetraosylceramide (Fig. 4B). As an orthogonal method, thin layer chromatography was used to visualize the most abundant glycosphingolipids extracted from rat eyes. We observed that signals co-migrating with the Gb3 standard were much more intense in glycosphingolipid extracts prepared from KO eye compared to WT eye (Fig. 4C). These thin layer chromatography and MS-based observations led us to conclude that KO rat eyes accumulate Gb3 to a significantly greater degree than WT rat eyes. Taken together, we provide evidence that Fabry rat eyes accumulate Gb3 and related α-galactosyl glycosphingolipids, which are important biomarkers that are elevated in patients.

**Figure 5:**
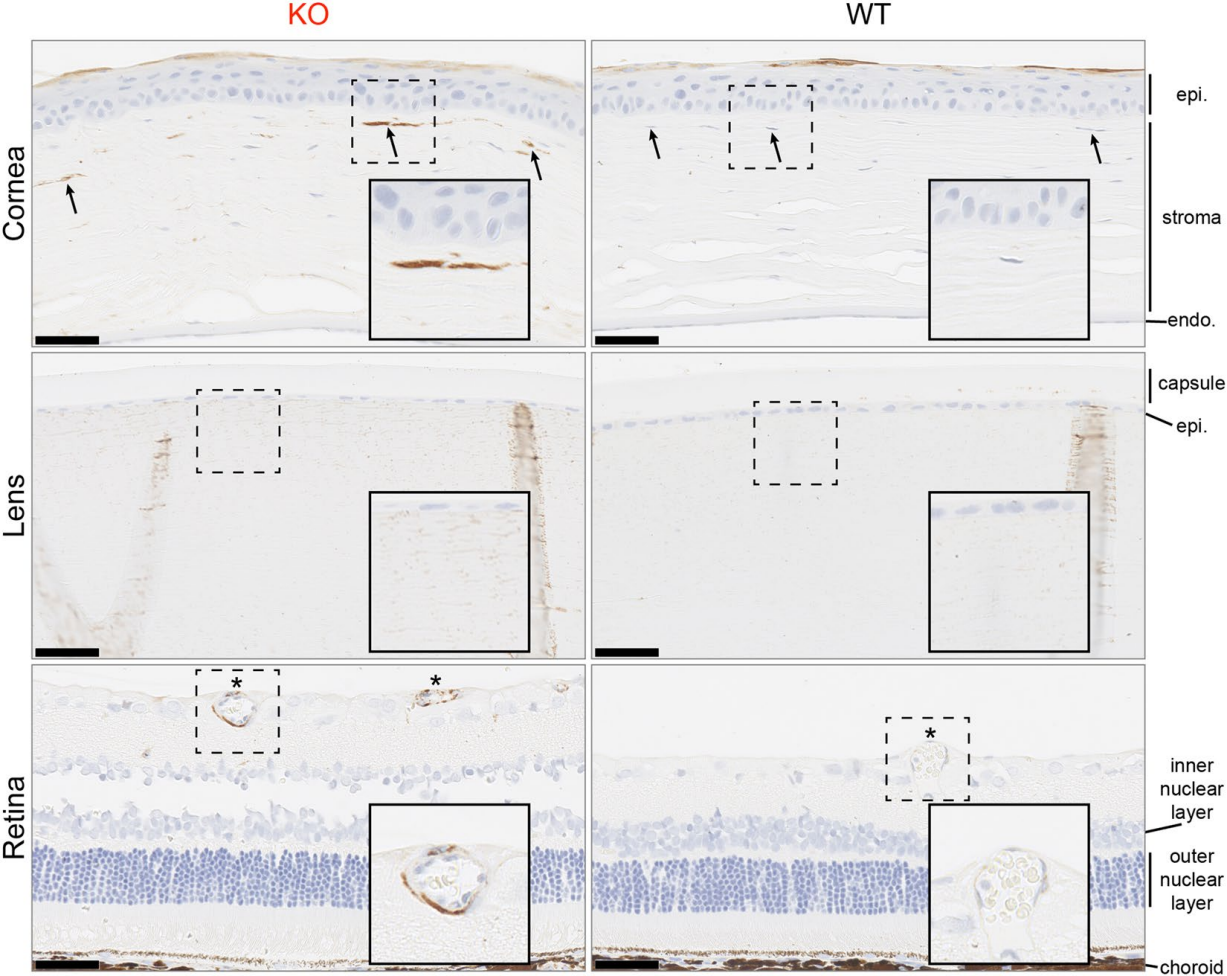
α-galactosyl glycoconjugates are detected in Fabry rat keratocytes, lens fibers, and retinal vessel endothelial cells. Rat eyes were fixed with 1% formalin/1.25% glutaraldehyde, and paraffin-embedded sections were stained with isolectin B4, a lectin that specifically binds to glycoconjugates with terminal α-galactose. Hematoxylin was used as a counterstain. Images are representative of 3 WT and 3 KO male rats at 80 weeks, and the scale bars represent 50 µm. Arrows indicate keratocytes, and asterisks indicate retinal vessels. The insets (solid boxes) show magnified views of the selected regions enclosed in dashed boxes. Abbreviations: epithelium (epi), endothelium (endo).

To qualitatively assess which ocular structures and cell types were susceptible to accumulation of the α-galactosyl glycosphingolipids, we stained rat eye sections with isolectin B4, which is specific for the terminal α-galactosyl epitope^18,19^. In other words, isolectin B4 staining marks the presence of α-galactosyl glycoconjugates. In the cornea, we found that KO keratocytes were strongly isolectin B4 positive, while WT keratocytes were isolectin B4 negative (Fig. 5, arrows). The isolectin B4-staining keratocytes had a predilection for the anterior cornea as opposed to the posterior cornea (Fig. 5). Less dramatic staining differences were observed in the lens, although KO lens fibers tended to stain with isolectin B4 to a greater degree than WT lens fibers (Fig. 5). The major finding in the retina was that the vascular endothelial cells stained with isolectin B4 in KO, but not WT, eye sections (Fig. 5, asterisks). To summarize, corneal keratocytes, lens fibers, and retinal vascular endothelial cells appear to be the locations of α-galactosyl glycoconjugate accumulation in Fabry rat eyes.

**Figure 6:**
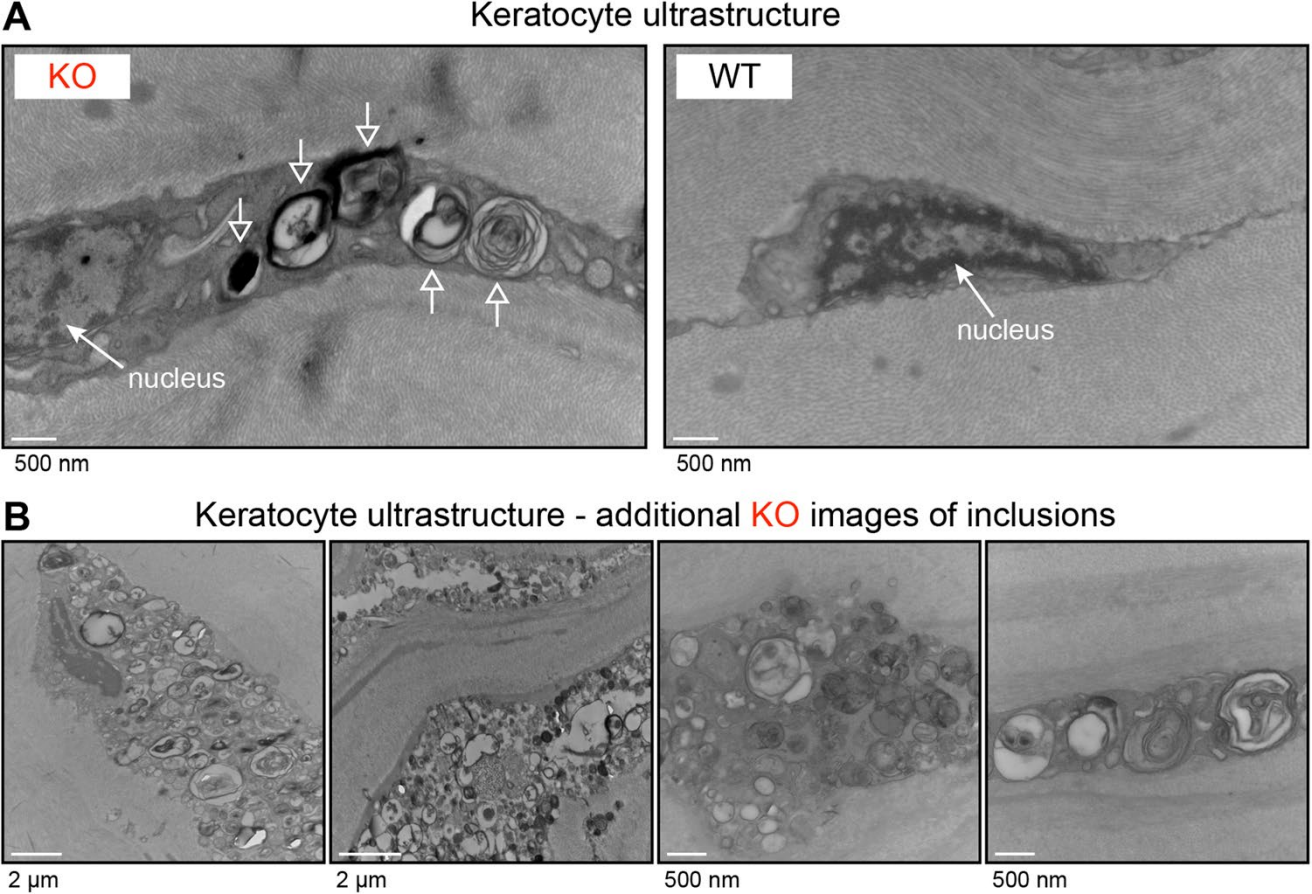
The ultrastructural morphologies of Fabry rat keratocyte inclusions are consistent with undigested storage material. (**A**) Shown are representative electron microscopy images of keratocytes from 2 WT and 2 KO rats between 57 and 63 weeks of age. Inclusions are indicated with the open arrows. (**B**) Additional electron microscopy images of the inclusion-laden keratocytes observed in KO rats are shown. Corresponding scale bar lengths are located below each image.

With the positive isolectin B4 staining of Fabry rat keratocytes, we lastly examined keratocyte ultrastructure. Electron microscopy was performed on approximately 60-week-old WT and KO rat corneas. We observed that KO keratocytes contained lamellar inclusions (Fig. 6A, arrows). Given that KO rat eyes accumulate α-galactosyl glycosphingolipids and KO rat keratocytes are isolectin B4 positive, these keratocyte inclusions are likely undigested α-galactosyl glycosphingolipids within lysosomes. Supporting this conclusion is the fact that lamellar inclusions are characteristic of lysosomal storage and are frequently observed in samples from patients with Fabry disease. No such inclusions were observed in WT keratocytes (Fig. 6A). The inclusions assumed diverse morphologies, and the storage burden appeared to cause keratocyte swelling (Fig. 6B). We conclude that Fabry keratocytes have a significant storage burden and likely underlie the development of the corneal opacities observed at the slit lamp biomicroscope (Fig. 1).

## Discussion

Patients with Fabry disease experience debilitating neuropathic pain and are at major risk of developing renal failure, heart disease, and cerebrovascular events (reviewed in^3^). These symptoms lead to a significantly compromised quality of life and shortened life span^20,21^. Because enzyme replacement therapy and chaperone therapy are currently available and novel treatments are on the horizon (reviewed in^22^), it is imperative that patients are diagnosed and treated as early as possible to prevent irreversible organ damage. The ocular manifestations of Fabry disease play an important role in diagnosis and may be useful in monitoring response to therapy. Although cornea verticillata does not impair vision, patients can have vision loss from cataracts and are at an increased risk of blinding retinal vascular occlusive events^7–9^. Further, the mechanisms behind ocular phenotype development are only now beginning to be explored. For example, Bitirgen and colleagues recently found evidence of corneal nerve damage in Fabry patients compared to healthy controls^23^. Therefore, an *in vivo* animal model that recapitulates Fabry ocular findings is required to both infer whether response to therapy can be monitored and to obtain mechanistic knowledge that complements human studies.

We aimed to systematically characterize the eyes of Fabry rats. Although we did not find evidence of retinal vascular tortuosity, we did demonstrate that retinal vascular abnormalities can occur. We also found that Fabry rats develop corneal and lenticular opacities to a significantly greater degree than age-, sex-, and littermate-matched WT controls. We demonstrated that whole eyes accumulate α-galactosyl glycosphingolipids and showed that these stored glycoconjugates are present in corneal keratocytes, lens fibers, and retinal vascular endothelial cells by histological staining. Further, ultrastructural examination revealed lamellar inclusion buildup in keratocytes, which is characteristic of glycosphingolipid storage. We conclude that Fabry rats develop many phenotypes observed in patients and can be used to study the pathogenesis and therapeutic monitoring capacity of these ocular lesions.

Data on ocular phenotypes in Fabry mice are lacking in the literature, and as such, our findings highlight a major advantage of the rat over the mouse in Fabry disease research. Ohshima and colleagues performed ophthalmic examinations on Fabry mice at 40 and 80 weeks; however, the authors reported that there were no differences compared with control mice^14^. Taguchi and colleagues examined the eyes of only one Fabry mouse and one Fabry mouse overexpressing Gb3 synthase. Punctate, lenticular opacities were observed in one eye of the Fabry mouse and in both eyes of the Fabry mouse overexpressing Gb3 synthase^24^. Corneal opacities, however, were not reported in either mouse in this limited ocular analysis. Further, the eyes of WT mice were not examined, making it impossible to determine whether the lenticular opacities were a consequence of α-Gal A deficiency or background strain. Using blinded scoring of α-Gal A deficient and WT rats, our report provides an advance in the ocular phenotyping of Fabry animal models as it robustly documents corneal and lenticular opacities in KO male and female rats in comparison to littermate WT rats.

Ocular findings are an important phenotype to assess because most patients develop ophthalmologic signs of Fabry disease. Cornea verticillata is found in approximately 85% of males and 75% of females, while lenticular opacities are found in approximately 30% of males and 10% of females^6^. Pitz and colleagues demonstrated that the ocular signs correlate well with disease severity in patients^4^. While cornea verticillata does not affect vision, it is a useful diagnostic sign for eye care professionals and can potentially be used to noninvasively monitor therapy response^7^. It is currently unknown whether ocular opacities stabilize or decrease with therapy. One recent case report demonstrated regression of corneal opacities in a patient after 16 years of enzyme replacement therapy^25^. As Fabry rats develop ocular opacities, this animal model can be used in future studies to answer the outstanding question of whether opacities regress with therapy. Other questions may be addressed, such as cellular distribution of various therapies and optimal age of therapy initiation to obtain maximal benefit. Fabry rats also have the potential to definitively determine whether ocular lesions serve as a noninvasive surrogate of therapeutic efficacy as the opacity scoring can be performed using a slit lamp microscope with the rat under anesthesia. If proven true, this would provide a non-invasive method and decrease the need for serial sacrifices in longitudinal, therapeutic efficacy experiments. Because these ophthalmological manifestations can be observed in living Fabry rats, they may be used as a valuable tool for evaluating the effectiveness of novel and existing therapeutics. Therefore, the ocular findings in Fabry rats have tremendous potential to inform future therapy studies.

In addition to its role in assessing therapeutic efficacy, cataract development in Fabry rats provides a model through which cataract biology in Fabry disease can be studied. A currently unanswered question is: why do Fabry rats and patients develop cataracts? Gb3 deposition likely does not directly result in opacities, but rather Gb3 accumulation leads to downstream dysfunction that leads to opacification. Three, non-mutually exclusive hypotheses surrounding the factors that promote Fabry cataract formation include: (1) dysfunction of lens epithelial cells, (2) changes in the profile of lens lipids, and (3) increased oxidation of lenticular lipids in Fabry rats compared to WT rats. Each hypothesis will be briefly expanded upon below. In order to explore these possibilities, future studies are needed to analyze the epithelial cells and lipids specifically in Fabry rat lenses (our current study analyzed lipids of whole eyes). The study of cataract formation in Fabry rats is of ophthalmological importance because these pathways can be targeted to prevent cataract development in Fabry patients and perhaps also in patients with age-related cataracts.

First, dysfunction of lens epithelial cells may lead to cataract development in Fabry disease. Lamellar aggregates have been observed in lens epithelial cells from a Fabry patient postmortem^26^. Because lens epithelial cells are responsible for maintaining lens transparency by producing α-crystallins (reviewed in^27^), lens epithelial cell dysfunction from lysosomal storage may explain cataract development in Fabry disease. For example, glycosphingolipid-laden lens epithelial cells may either have impaired synthesis and secretion of α-crystallins or may be more prone to cell death, inhibiting their ability to preserve lens transparency. Thus, compromised lens epithelial cells may contribute to a cataractous lens.

Second, lens lipid changes, such as accumulation of α-galactosyl glycosphingolipids may lead to cataract development. In humans, the percent sphingolipid content is known to be much higher in opaque lenses compared to clear lenses (reviewed in^28^). Further, gangliosides have been shown to increase with cataract progression^29^. Given that sphingolipids and gangliosides play established roles in cataracts, an increase in α-galactosyl glycosphingolipids within the lens may similarly lead to cataract development in Fabry patients. Future studies are needed to explore the lipid profiles of Fabry rat lenses as this study only documented α-galactosyl glycosphingolipid accumulation in whole rat eyes.

Third, increased oxidation of lenticular lipids may lead to cataract development. In the lens, approximately 90% of total oxygen is consumed by mitochondrial respiration^30^, indicating that normal mitochondrial function is essential in controlling the lenticular oxidative environment. In fibroblasts from patients with Fabry disease, the activities of respiratory chain enzymes I, IV, and V are reduced^31^. This may be a result of lysosomal dysfunction, where damaged mitochondria persist due to impaired mitophagy. Decreased mitochondrial activity may also occur in lens epithelial cells in Fabry disease, leading to an increased burden of oxygen and reactive oxygen species, resulting in the formation of a cataract. To summarize, knowledge of the mechanisms contributing to cataract development in Fabry disease will not only be useful in treating Fabry patient lens opacities but may also be translatable in treating those with age-related cataracts.

We did not observe macroscopic retinal tortuosity in the KO rats, but we did observe microscopic abnormalities in the retinal vasculature of KO rats, such as accumulation of α-galactosyl glycoconjugates within the retinal vascular endothelial cells. Storage of glycosphingolipids within the vascular endothelial cells may be a precursor to vascular occlusive disease seen in patients, resulting in blindness or neovascularization. In addition, the one KO rat with vascular leakage on fluorescein angiography may be a demonstration of vascular compromise from endothelial cell dysfunction.

We hypothesized that α-galactosidase A deficiency in rats would lead to corneal, lenticular, and retinal phenotypes similar to those observed in patients with Fabry disease. We found that Fabry rats develop corneal and lenticular opacities, but not retinal vascular tortuosity. We quantified α-galactosyl glycosphingolipid biomarkers in whole eye, and we established that these glycosphingolipids are located in corneal keratocytes, lens fibers, and retinal vascular endothelial cells. We conclude that the characterization of ocular findings in Fabry rats provides the foundation by which ocular pathogenesis can be studied. Further, this rat model can be used to determine whether therapeutic efficacy can be noninvasively evaluated using the observed ocular abnormalities.

## Supplementary information

Supplementary information

## Data Availability

The datasets generated during in this study are available from the corresponding author on reasonable request.
