# Supplementary material for: Rats deficient in α-galactosidase A develop ocular manifestations of Fabry disease
Source: Sci Rep. 2019 Jun 28;9:9392. doi: 10.1038/s41598-019-45837-1 (PMC6599056; doi:10.1038/s41598-019-45837-1)
Supplement: Supplementary file 1 — Supplementary information [file 41598_2019_45837_MOESM1_ESM.docx]

**Supplemental information:**

**Rats deficient in α-galactosidase A develop ocular manifestations of Fabry disease**

James J. Miller^1^, Kazuhiro Aoki^2^, Christopher A. Reid^3^, Michael Tiemeyer^2^, Nancy M. Dahms^1*^, Iris S. Kassem^3,4*^

^1^Department of Biochemistry, Medical College of Wisconsin, Milwaukee, WI

^2^Complex Carbohydrate Research Center, University of Georgia, Athens, GA

^3^Department of Cell Biology, Neurobiology, and Anatomy, Medical College of Wisconsin, WI

^4^Department of Ophthalmology and Visual Sciences, Medical College of Wisconsin, WI

*Correspondence: Iris S. Kassem, MD, PhD, Department of Ophthalmology and Visual Sciences and Department of Cell Biology, Neurobiology, and Anatomy, Medical College of Wisconsin Eye Institute, 925 N. 87th Street, Milwaukee, WI 53226, Phone: 414-955-7803, Fax: 414-955-6300, Email: [ikassem@mcw.edu](mailto:ikassem@mcw.edu), ORCID identifier: https://orcid.org/0000-0002-3351-9779; Nancy M. Dahms, PhD, Department of Biochemistry, Medical College of Wisconsin, 8701 W. Watertown Plank Rd., Milwaukee, WI 53226, Phone: 414-955-4698, Fax: 414-955-6510, Email: [ndahms@mcw.edu](mailto:ndahms@mcw.edu), ORCID identifier: https://orcid.org/0000-0001-6819-0590

**
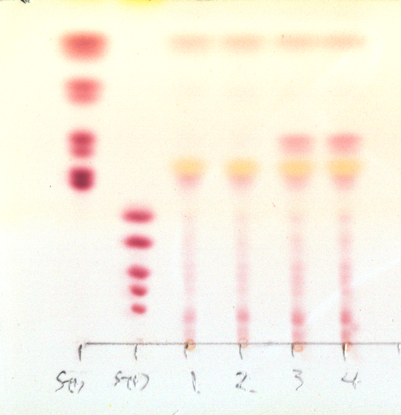
**

**Supplemental Figure 1: Full image of thin layer chromatography plate in Figure 4C.** “STD” refers to lanes where glycosphingolipid standards were spotted. Lanes 1 and 2 refer to samples from WT rats and lanes 3 and 4 refer to samples from KO rats (as shown in Figure 4C).
